# Phytoceramide and sphingoid bases derived from brewer's yeast *Saccharomyces pastorianus *activate peroxisome proliferator-activated receptors

**DOI:** 10.1186/1476-511X-10-150

**Published:** 2011-08-24

**Authors:** Itsuo Murakami, Yukari Wakasa, Shinji Yamashita, Toshio Kurihara, Kota Zama, Naoyuki Kobayashi, Yukiko Mizutani, Susumu Mitsutake, Tatsuro Shigyo, Yasuyuki Igarashi

**Affiliations:** 1 Department of Biomembrane and Biofunctional Chemistry, Faculty of Advanced Life Sciences, Hokkaido University, Nishi 11, Kita 21, Kita-ku, Sapporo 001-0021, Japan; 2 Frontier Laboratories of Value Creation, Sapporo Breweries, Ltd., 10 Okatohme, Yaizu, Shizuoka 425-0013, Japan

**Keywords:** PPARs, phytoceramide, brewer's yeast

## Abstract

**Background:**

Peroxisome proliferator-activated receptors (PPARs) are ligand-activated transcription factors that regulate lipid and glucose metabolism. PPARα is highly expressed in the liver and controls genes involved in lipid catabolism. We previously reported that synthetic sphingolipid analogs, part of which contains shorter-length fatty acid chains than natural sphingolipids, stimulated the transcriptional activities of PPARs. Sphingosine and dihydrosphingosine (DHS) are abundant sphingoid bases, and ceramide and dihydroceramide are major ceramide species in mammals. In contrast, phytosphingosine (PHS) and DHS are the main sphingoid bases in fungi. PHS and phytoceramide exist in particular tissues such as the epidermis in mammals, and involvement of ceramide species in PPARβ activation in cultured keratinocytes has been reported. The purpose of the present study is to investigate whether natural sphingolipids with C18 fatty acid and yeast-derived sphingoid bases activate PPARs as PPAR agonists.

**Method:**

Lipids of brewer's yeast contain PHS- and DHS-based sphingolipids. To obtain the sphingoid bases, lipids were extracted from brewer's yeast and acid-hydrolyzed. The sphingoid base fraction was purified and quantified. To assess the effects of sphingolipids on PPAR activation, luciferase reporter assay was carried out. NIH/3T3 and human hepatoma (HepG2) cells were transfected with expression vectors for PPARs and retinoid × receptors, and PPAR responsive element reporter vector. When indicated, the PPAR/Gal4 chimera system was performed to enhance the credibility of experiments. Sphingolipids were added to the cells and the dual luciferase reporter assay was performed to determine the transcriptional activity of PPARs.

**Results:**

We observed that phytoceramide increased the transcriptional activities of PPARs significantly, whereas ceramide and dihydroceramide did not change PPAR activities. Phytoceramide also increased transactivation of PPAR/Gal4 chimera receptors. Yeast-derived sphingoid base fraction, which contained PHS and DHS, or authentic PHS or DHS increased PPAR-dependent transcription. Additionally, phytoceramide stimulated PPARα activity in HepG2 hepatocytes, suggesting that phytoceramide activates genes regulated by PPARα.

**Conclusions:**

Phytoceramide and yeast-derived sphingoid bases activate PPARs, whereas ceramide and dihydroceramide do not change the PPAR activity. The present findings suggest that phytoceramide acts as a PPAR ligand that would regulate PPAR-targeted genes.

## Background

Peroxisome proliferator-activated receptors (PPARs) are members of nuclear hormone receptors that act as ligand-activated transcription factors. PPARs regulate expression of genes involved in lipid and glucose metabolism [1,2]. PPARs consist of three isoforms, PPARα, PPARβ (also known as PPARδ), and PPARγ. PPARα is expressed at high levels in liver, where activated PPARα promotes fatty acid oxidation and ketogenesis. PPARβ is expressed ubiquitously and is implicated in lipid catabolism and energy production in adipose tissue and skeletal muscle [3]. PPARβ is also involved in epithelial cell differentiation and wound healing in the epidermis [4]. PPARγ is highly expressed in white adipocytes where it stimulates the intake of fatty acids and glucose and the synthesis of triglycerides [5].

**Figure 1:**
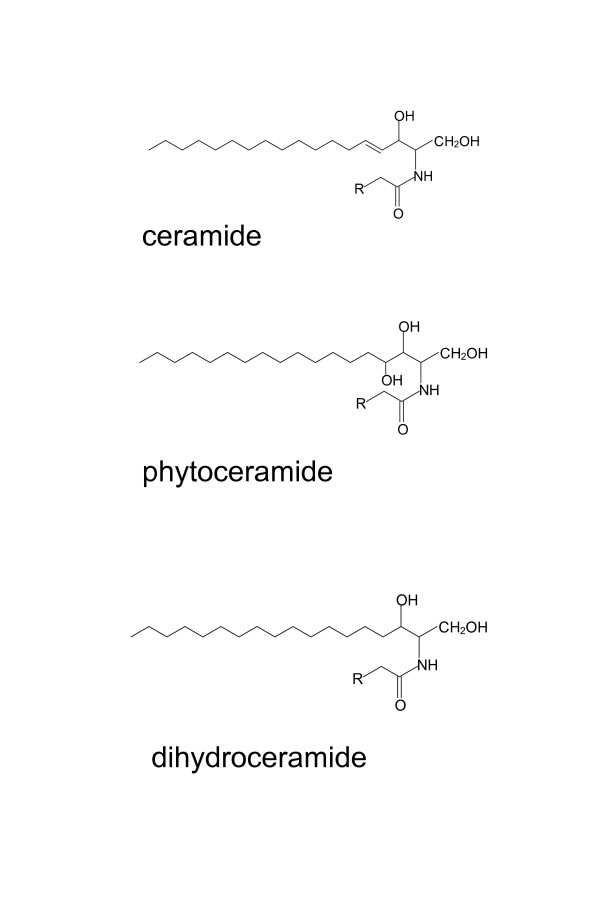
**Chemical structure of ceramide species (ceramide, phytoceramide and dihydroceramide)**.

We previously reported that synthetic sphingolipid analogs activated PPARs when assessed using the luciferase reporter system [6]. This observation gave rise to the speculation that natural sphingolipids may act as PPAR ligands. Ceramide is the basic structure of complex sphingolipids, which are present in the plasma membrane. It is composed of a long-chain sphingoid base with the 2-amino group amide-linked to a fatty acid [7,8]. The fatty acids are commonly saturated or mono-unsaturated, and their chain length varies from 14 to 26. In mammalian cells, sphingolipids contain a sphingoid base, mainly sphingosine (Sph) or dihydrosphingosine (DHS). The major type of sphingoid base, Sph, has a double bond between C4 and C5 position, whereas DHS is saturated at that site (Figure 1). Phytosphingosine (PHS), which has hydroxyl group at the C4 position, exists in certain tissues, in particular the intestine, kidney and epidermis of mammals [9-11]. All sphingolipid analogs that activated PPARs [5] had a PHS-based structure. In the mammalian epidermis, sphingolipids play a critical role in water retention and in creating the permeability barrier against extraneous substances [11,12]. Expression of DES2, a hydroxylase enzyme that produces phytoceramide from dihydroceramide, is upregulated during the differentiation of cultured keratinocytes [13]. It has been reported that PPARβ expression and activation brought about keratinocyte differentiation in response to inflammatory signals, such as tumor necrosis factor-α and interferon-γ, and that exogenous administration of short-chain ceramides enhanced PPARβ activity, which increased the cells' resistance to apoptosis [14]. These previous studies suggest that ceramide species are responsible for PPARβ activation during keratinocyte differentiation.

In contrast to mammals, yeasts contain mainly two sphingoid bases: PHS and DHS. Brewer's yeast is a member of budding yeasts, and residues of brewer's yeast after beer manufacturing contain sphingolipids including mainly mannosyl-diinositolphosphoryl-ceramide [M(IP)_2_C] [15,16].

Sphingolipids are found in many foodstuffs [17,18], but the effect of dietary sphingolipids on PPAR activity is not understood. In this study, we examine whether ceramide species with C18 fatty acid, which is present in mammals, act as PPAR ligands, using the luciferase reporter assay system. We also extracted sphingoid bases from brewer's yeast residues and assessed their effect on PPAR activity.

## Materials and methods

### Materials

C18 phytoceramide, C18 ceramide, C18 dihydroceramide, C2 ceramide, PHS and DHS were purchased from Avanti polar lipids (Alabaster, AL, USA). WY-14643 (WY), L-165, 041 (LD) and ciglitazone (CIG) were obtained from Sigma-Aldrich (St. Louis, MO, USA). C18 phytoceramide, C18 ceramide and C18 dihydroceramide were dissolved in chloroform/methanol (2:1 [vol:vol]), and then were dried under reduced pressure using a Speed Vac concentrator. The dried samples were dissolved in DMSO, and used in treatment of the cells. The sphingoid bases were dissolved in EtOH and were employed for cell treatment except as indicated in the figure legends.

### Preparation of sphingoid bases from brewer's yeast

Lipids were extracted from brewer's yeast, and saponified with 1 N NaOH in MeOH. The lipids were acid-hydrolyzed with 9.4% H_2_O, 27.3% MeOH, 54.7% tetrahydrofuran, and 1 N HCl for 18 h at 60°C. After hydrolysis, an equal volume of hexane was added, then the samples were centrifuged and the hexane phase was removed. Partitioning was repeated another two times. To one volume of sample half volume of 2 N NaOH and equal volume of chloroform were added, and the mixture was centrifuged for separation. The lower chloroform phase was collected and washed twice with Folch's upper phase solution, and the chloroform phase was dried by evaporation. Dried samples were dissolved in 0.1 N NaOH and MeOH, 4:3 v/v, and sonicated in a sonication bath, and an equal volume of chloroform was added for partition. The upper aqueous phase was removed and the lower chloroform phase was washed twice by addition of an equal amount of Folch's upper phase. The washed lower phase was dried, and desalted using Sep-pak Plus C18 cartridge (Waters, Milford, MA, USA), and then the eluted sphingoid bases were used for cell treatments. A small portion of samples were used for high performance liquid chromatography (HPLC) analysis. Samples were dissolved in 120 μl of EtOH and heated for 15 min at 60°C, then mixed with 15 μl of OPA reagent (1 mg/ml o-phthalaldehyde, 0.2% v/v 2-mercaptoethanol in 3%, w/v, boric acid solution adjusted to pH 10.5) and incubated at 60°C for 1 h. After centrifugation at 10,000 g for 5 min, the supernatants were resolved by HPLC (Shimazu LC-10AD series; Shimazu, Kyoto, Japan) on a pre-packed C18 reversed-phase column (Cosmosil 5C18-AR-II; Nacalai Tesque, Kyoto, Japan) using an isocratic eluent composition of acetonitrile/distilled water (85:15, v/v) and a flow rate of 1 ml/min. The OPA derivatives were monitored at an excitation wavelength of 340 nm and an emission wavelength of 455 nm.

### Luciferase reporter assay

NIH/3T3 and HepG2 cells were cultured in Dulbecco's modified Eagle's medium (DMEM, Sigma-Aldrich) containing 2 mM L-glutamine and 25 mM glucose supplemented with 10% (v/v) heat-inactivated fetal calf serum (Invitrogen, Carlsbad, CA, USA) and antibiotics (100 units/ml penicillin and 0.1 mg/ml streptomycin) (Sigma-Aldrich) at 37°C in a humidified atmosphere of 5% CO_2_-95% air. For luciferase measurement, cells were plated in 96-well plates (BD Biosciences, Bedford, MA, USA) at 1 × 10^4 ^cells and allowed to adhere for 24 h. Cells were transfected using Lipofectamine 2000 (Invitrogen) at a ratio of 2.5 μl: 1 μg DNA, according to the manufacturer's instructions. Each well contained 10 ng of full-length mouse PPAR (pCMX-PPARα, PPARβ, or PPARγ), 10 ng of pCMX-RXR (retinoid × receptor), 160 ng of 4 × PPRE (PPAR responsive element) reporter plasmid (a reporter plasmid) and 20 ng of pGL4 TK Renilla luciferase plasmid (an internal control for normalizing transfection efficiency). For luciferase assay using the Gal4/PPAR chimera system, 150 ng of pG5 luc vector that contained Gal4 upstream activating sequences (UAS), and 50 ng of pBIND mouse PPARα, PPARβ or PPARγ (an expression plasmid for fusion protein of Gal4 DNA-binding domain and PPAR ligand binding domain (LBD)) were transfected into NIH/3T3 cells. For AP-1 activity measurement, 180 ng of 6 × AP-1 reporter plasmid and 20 ng of pGL4 TK Renilla luciferase plasmid were transfected to NIH/3T3 cells. Twenty-four hours after transfection, test compounds were diluted with phenol red-free DMEM (Invitrogen) containing 10% (v/v) charcoal-stripped fetal calf serum (cFCS) and were added to each well. After 24 h, the cells were lysed and assayed using a Dual-Glo Luciferase Assay System (Promega, Madison, WI, USA) according to the manufacturer's protocol.

### Cell viability analysis

NIH/3T3 cells were plated in a 96-well plate at a density of 1 × 10^4 ^cells per well. Cells were treated with phenol red-free DMEM (with 10% cFCS) containing indicated compounds. Cell viability was assessed at 24 h post-treatment using cell counting kit-8 (Dojin Chemical, Kumamoto, Japan), following the manufacturer's protocol.

### Statistical Analysis

Data are presented as means ± standard deviation (S.D.) of three samples. Statistical analysis was performed using an unpaired Student's t-test or one way ANOVA followed by Dunnett's test. Statistical significance was established at P < 0.05, 0.01 or 0.001 versus vehicle control. Each experiment was performed at least three times and produced consistent results.

## Results

### Phytoceramide activates PPARs

**Figure 2:**
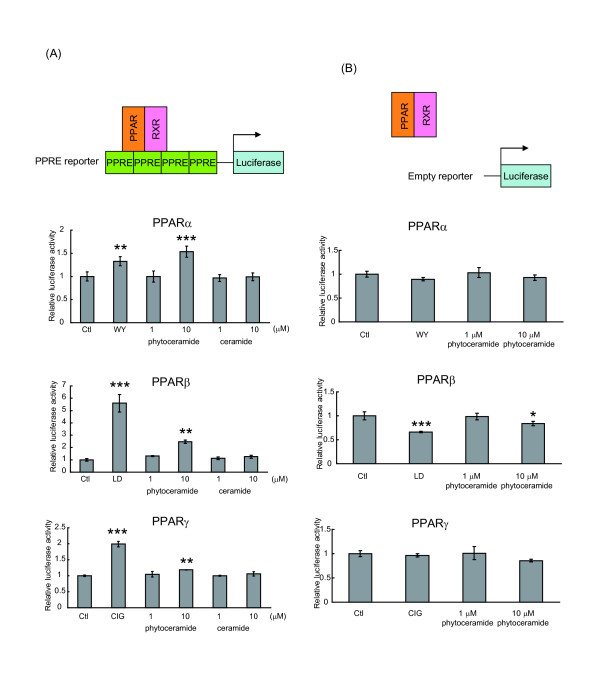
**Effects of phytoceramide on PPAR transcriptional activity**. (A) NIH/3T3 cells were transfected with 4 × PPRE firefly luciferase reporter plasmid along with expression plasmids for PPARs (PPARα, PPARβ or PPARγ) and RXR and an internal control TK Renilla luciferase vector. After 24 h cultivation, the cells were treated with the indicated concentration of phytoceramide or ceramide for 24 h. As positive control, 10 μM of WY-14643 (WY), L-165,041 (LD) or ciglitazone (CIG) were used. (B) NIH/3T3 cells were transfected as described above, expect for an empty luciferase reporter instead of 4 × PPRE firefly luciferase reporter. Twenty-four hours after transfection, phytoceramide was added to the cells for 24 h. The luciferase activity of the cell was measured, and firefly luciferase activity was normalized to Renilla luciferase activity. The activity of a vehicle control was expressed as 1 and the relative luciferase activities were presented as a fold induction to that of the vehicle control. All results are shown as means ± S.D. (n = 3). *P < 0.05, **P < 0.01, ***P < 0.001, significantly different from the level of vehicle control (Dunnett's test).

The sphingolipid analogs that activated PPARs had a PHS-based structure and a part of them had shorter-length fatty acid chains than natural ceramide species [6]. We first tested whether phytoceramide with C18 fatty acid, which exists in mammalian cells, activates PPARs. Phytoceramide at 10 μM significantly increased PPAR activity in NIH/3T3 cells (Figure 2A). PPARα and PPARγ activities induced by 10 μM phytoceramide were increased by 1.5-fold and 1.2-fold, respectively. PPARβ was more intensely activated in the presence of 10 μM phytoceramide (2.5-fold) than PPARα and PPARγ. WY, LD and CIG, which are synthetic PPARα, PPARβ and PPARγ ligands, respectively, are used as positive control reagents. In contrast, C18 ceramide did not show significant PPAR activation. In the control experiments for evaluating the specificity of PPAR activation, the luciferase activity of the pGL4.23 empty vector that did not contain the PPRE sequence was not changed by phytoceramide treatment in PPARα- and PPARγ-transfected cells (Figure 2B). In PPARβ-transfected cells, LD and 10 μM phytoceramide treatment decreased luciferase activity of the empty reporter, probably owing to decreased background transcription of luciferase. Taken together, these results indicate that phytoceramide activates PPARs in a PPRE-dependent manner, but ceramide does not change the activities.

**Figure 3:**
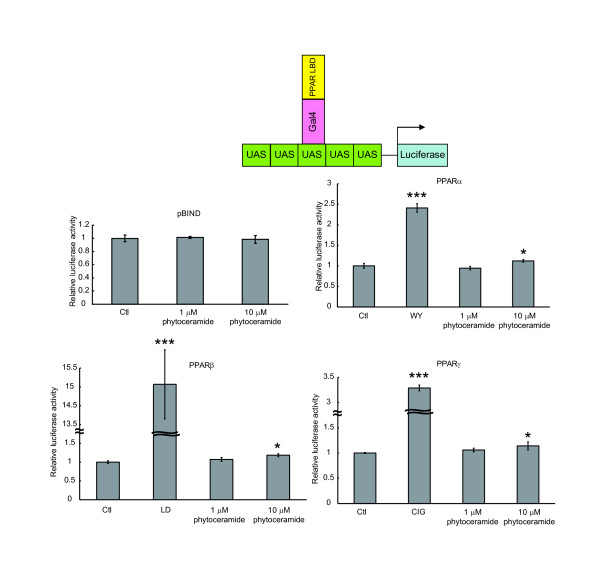
**Effect of phytoceramide on PPAR activity in the Gal4/PPAR chimera system**. The expression vector for PPAR LBD/Gal4 DBD and Gal4 UAS luciferase reporter were transfected into NIH/3T3 cells. After 24 h, cells were treated with phytoceramide at the indicated concentrations. The luciferase assay was performed as described in the legend to Figure 2A. All results are shown as means ± S.D. (n = 3). *P < 0.05, ***P < 0.001, significantly different from the level of vehicle control.

To confirm the accuracy of the PPAR activation by addition of phytoceramide, we employed another reporter system in which the LBD of PPARs was fused to a DNA-binding domain of the yeast transcription factor Gal4 [19]. The chimera receptor can bind to Gal4 UAS and activate transcription when ligands bind to PPAR LBD. This method is independent of endogenous PPARs and specific for ligand binding to PPARs [20]. Figure 3 shows that the transcriptional activation by exposure to phytoceramide at 10 μM depends on the presence of PPAR LBD. At this concentration, the activities of PPARα, PPARβ and PPARγ induced by phytoceramide increase about 1.1-, 1.2- and 1.1-fold, respectively, whereas that of the pBIND parental vector alone (Gal4 only) was not changed. In this assay system, the degree of activation of PPARβ was higher than that of PPARα and PPARγ, similarly to native PPARs (Figure 2A).

All these results obtained from different reporter systems indicate that phytoceramide activates PPARs, especially PPARβ, suggesting that not only synthetic sphingolipid analogs but also phytoceramide acts as an agonist of PPARs.

### Effect of yeast-derived sphingoid bases in PPAR activity

**Figure 4:**
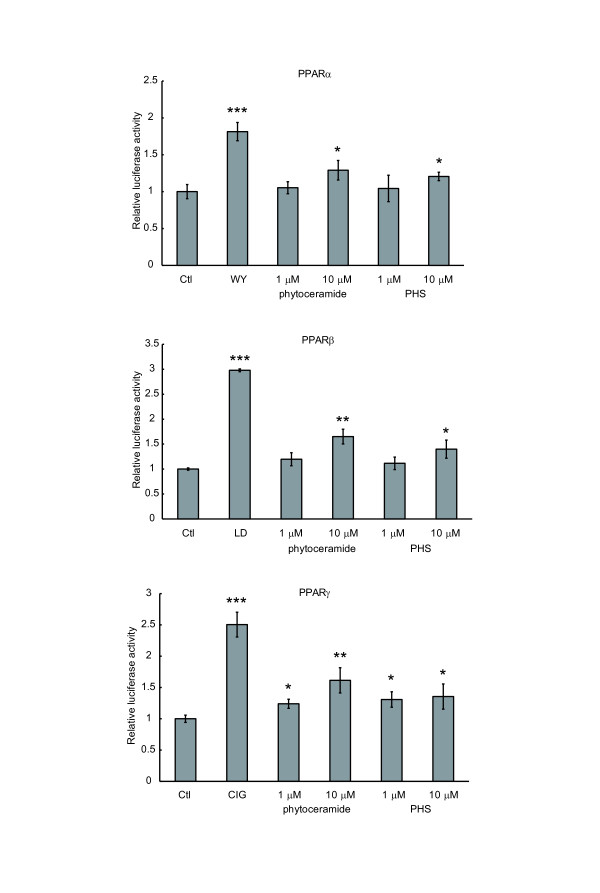
**Comparison between phytoceramide and PHS in PPAR activation**. NIH/3T3 cells were transfected as described for Figure 2A. PHS was dissolved in DMSO to carry out the experiments under the same condition with phytoceramide. Transfected cells were treated with phytoceramide or PHS at the indicated concentrations for 24 h, and then the luciferase activity was quantified. All results are shown as means ± S.D. (n = 3). *P < 0.05, **P < 0.01, ***P < 0.001, significantly different from the level of vehicle control.

**Figure 5:**
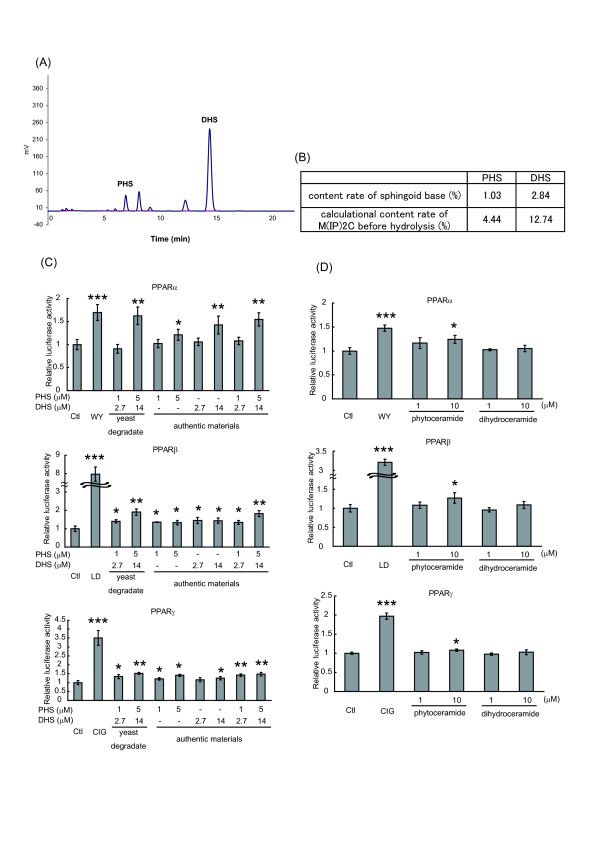
**Effects of sphingoid bases derived from brewer's yeast in PPAR activation**. (A and B) Sphingoid base fraction from brewer's yeast was extracted, treated with o-phthalaldehyde, and analyzed by reversed-phase HPLC. The areas of PHS and DHS were quantified. (C) NIH/3T3 cells were transfected as described for Figure 2A. Cells were stimulated with the indicated concentration of yeast-derived sphingoid base fraction. Cells were also treated with authentic PHS, DHS or the mixture of PHS and DHS. (D) Transfected cells were cultured with phytoceramide or dihydroceramide (1 or 10 μM) for 24 h. Luciferase activity was presented as a fold induction relative to that of the control. All results are shown as means ± S.D. (n = 3). *P < 0.05, **P < 0.01, ***P < 0.001, significantly different from the level of vehicle control.

Ceramides are degraded to a sphingoid base and a fatty acid by ceramidase. Thus, the possibility arose that PPAR activation by phytoceramide might be caused by PHS generated from phytoceramide. As shown in Figure 4, 10 μM PHS induced a 1.2-, 1.4- and 1.3-fold activation of PPARα, PPARβ and PPARγ, respectively, while 10 μM phytoceramide caused a 1.3-, 1.6- and 1.6-fold activation of PPARα, PPARβ and PPARγ, respectively, indicating that PHS also activated PPARs. Since yeast sphingolipids have a PHS-based ceramide backbone, we next investigated the effect of sphingolipids obtained from brewer's yeast on PPAR activation. M(IP)_2_C has a hydrophilic head group moiety. Thus, the cells will be hard to uptake and utilize it. To remove the hydrophilic head group moiety, we hydrolyzed the lipids from yeast extracts, purified them and obtained a sphingoid base fraction. The purified sphingoid base fraction was analyzed by HPLC. Peaks corresponding to PHS and DHS were observed at 1.03% and 2.84% of the extracted lipid, respectively (Figure 5A and 5B). Original amounts of M(IP)_2_C with PHS or DHS before acid hydrolysis were estimated from this measurement. The sphingoid bases were added to NIH/3T3 cells at the concentration of 1 μM PHS and 2.7 μM DHS (low concentration) or 5 μM PHS and 14 μM DHS (high concentration), in comparison with the same concentrations of authentic PHS and DHS (Figure 5C). Yeast-extracted sphingoid bases increased PPAR activities, and the mixtures of authentic PHS and DHS raised the activities of PPARs comparably to the equivalent concentrations of yeast-derived sphingoid bases. Intriguingly, the activities of PPARs were stimulated by DHS alone as well as PHS alone. We then examined the effect of dihydroceramide, which was composed of DHS with a fatty acid. In NIH/3T3 cells dihydroceramide did not show any effect on transactivation of PPARs under the same conditions as phytoceramide treatment (Figure 5D). These results indicate that yeast-derived sphingoid bases can activate PPARs, and that either PHS and/or DHS similarly stimulate transcriptional activities of PPARs but dihydroceramide does not alter PPAR activities. It was considered that the PHS and/or DHS were responsible for PPAR activation by yeast-derived sphingoid bases.

### Activation of PPARs is not caused by alteration of cell viability

**Figure 6:**
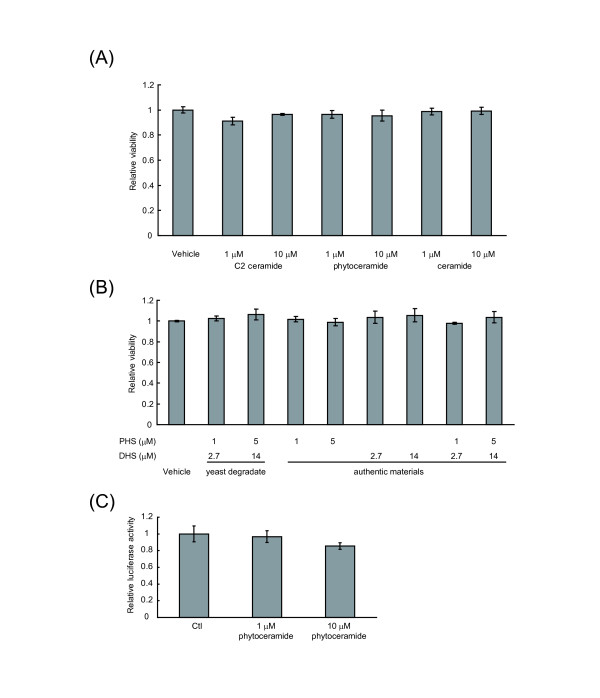
**Effects of sphingolipid administration on cell viability**. NIH/3T3 cells were seeded into a 96-well culture plate. (A) C2 ceramide, phytoceramide and ceramide were dissolved in DMSO, and (B) yeast degradate, PHS and DHS were dissolved in EtOH. These sphingolipids were added to the cells for 24 h. Cell viability was measured using Cell Counting Kit-8. Cell viability is expressed as a relative value to vehicle control. (C) NIH/3T3 cells were transfected with 6 ×AP-1 reporter and TK Renilla luciferase vector for 24 h. Cells were treated with the indicated concentration of phytoceramide for 4 h. The luciferase activity was measured and expressed as fold change of control (Ctl). The results represent the means of triplicate determinations ± S.D. from a representative experiment.

It has been reported that exogenous administration of ceramide exhibited a cytotoxic effect [21,22], and that ceramide-generated endogenous ligand of PPARβ increased its activity via AP-1 pathway in keratinocytes [14]. To investigate the possibility that the increased luciferase activities by sphingolipid treatment could be due to the secondary cytotoxic effect, cell viabilities in the presence of sphingolipids were tested. C2 ceramide, which was reported as an apoptotic inducer, exhibited no alteration in the viability of NIH/3T3 cells in our experiment (Figure 6A). Similarly, cell viability was not changed by the addition of phytoceramide, ceramide, dihydroceramide or the yeast-derived sphingoid bases, PHS and DHS. In addition, phytoceramide did not change the AP-1 activity in NIH/3T3 cells (Figure 6B). These results suggest that PPAR activation by sphingolipids is not due to the results of decreased cell viability and AP-1 activation.

### Phytoceramide activates PPARα in hepatocytes

**Figure 7:**
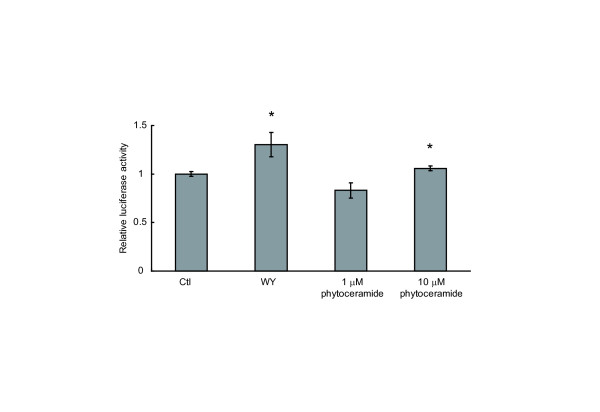
**Effect of phytoceramide on transcriptional activity of PPARα in HEPG2 hepatocytes**. HEPG2 cells were transfected with 4 × PPRE reporter plasmid and expression vectors for PPARα and RXR. TK Renilla luciferase reporter was cotransfected for normalizing the transfection efficiency. Following transfection, cells were treated for 24 h with phytoceramide (1, 10 μM). Luciferase assays were performed as described elsewhere. All results are shown as means ± S.D. (n = 3). *P < 0.05, significantly different from the level of vehicle control.

PPARα is highly expressed in the liver where it has been shown to promote fatty acid oxidation. Fibrate drugs function as agonists of PPARα and have been used for the treatment of hypertriglyceridemia [23]. To elucidate whether phytoceramide induces PPARα activation in hepatocytes, human hepatoma cells, HepG2, were used instead of NIH/3T3 (Figure 7). Phytoceramide at 10 μM caused a 1.1-fold induction of PPARα activity. This result suggests that phytoceramide that was derived from daily diets and reached the liver could stimulate PPARα and regulate PPARα target genes in the liver.

## Discussion

In the present study, we found that phytoceramide activated PPARs and that PPARβ was more highly stimulated than PPARα and PPARγ. However, ceramide and dihydroceramide did not activate PPARs (Figure 2A and 5D). Analysis of the X-ray structure indicated a hydrogen bond between the ligand and LBD residues of human PPARγ LBD [24,25]. The hydroxyl group at C4 position in phytoceramide is not present in ceramide and dihydroceramide; therefore, the hydroxyl group at C4 position might be involved in the transcriptional activation of PPARs. Another hypothesis that could explain the superiority of phytoceramide in PPAR activation is that phytoceramide is more easily taken up into the cells than dihydroceramide and ceramide. In that case, the structural difference among ceramide species might have no correlation with PPAR activation.

Phytoceramide is synthesized mainly in the kidney, intestine and skin in mammals, while PHS and DHS are the major sphingoid bases in fungi. Fermented foods contain the PHS-based sphingolipids derived from fungi, and a some of the sphingolipids are hydrolyzed to sphingoid bases in the small intestine [26]. The sphingoid bases are taken up by intestinal cells and reincorporated into complex sphingolipids such as ceramide and sphingomyelin. Some of them reach the lymph, blood and liver [26,27]. We observed that PHS contained in food was absorbed and reached the mouse liver (unpublished data). We also observed that phytoceramide induced activation of PPARα in HepG2 hepatocytes (Figure 7). Therefore, phytoceramide originating from dietary yeast sphingolipids could be incorporated to the cells, such as hepatocytes and adipocytes, and activate PPARs, regulating the expression of PPARα target genes.

In addition to phytoceramide, yeast-derived sphingoid bases and authentic PHS and DHS treatment activated PPARs (Figure 5C), while ceramide and dihydroceramide treatment did not activate PPARs. There were no distinct differences between yeast-derived sphingoid bases and authentic sphingoid bases in PPAR activation, but their effects were smaller than for the positive controls (WY, LD and CIG) (Figure 5C). These results indicate that activation of PPARs by yeast-derived sphingoid bases does not reach maximal levels, suggesting that unidentified factors included in yeast-derived sphingoid bases do not contribute to PPAR activation. There is a possibility that PHS and DHS interact directly with PPARs and activate PPARs. The incorporation rate of sphingoid bases into the cells is higher than that of ceramide species [28,29]. If PHS was a true ligand of PPARs, PPAR activation occurring by PHS treatment would be higher than phytoceramide treatment. However, the activation of PPARs by PHS was not more potent than phytoceramide at the same concentration, as shown in Figure 4. Therefore, we considered that PHS-induced activation of PPARs was caused by phytoceramide generated from PHS in the cells. It remains unclear why DHS treatment activates PPARs or whether metabolites of DHS are the active agents. Future studies are needed to elucidate this question.

In this study, PPARβ was most intensively stimulated by phytoceramide in three PPARs. It has been reported that liver-specific PPARβ overexpression decreases glucose production and increases glucose disposal in the liver [30]. Moreover, transgenic mice overexpressing constitutively active PPARβ in white adipocytes exhibit reduced fat mass [31]. Therefore, phytoceramide-induced activation via PPARβ might contribute to the anti-obesity effect.

It has been reported that PPARβ is concerned with keratinocyte differentiation, and the DES2 protein that converts dihydroceramide to phytoceramide is expressed in differentiated keratinocytes. Glucosylceramide in the extracellular spaces of the epidermis is hydrolyzed to free ceramide species [32], and these lipids are involved in skin barrier function. Alkaline ceramidase is highly expressed in mouse skin and does not digest phytoceramide well [33]. Therefore, there is a possibility that phytoceramide in epidermis is protected from digestion and that phytoceramide might regulate epidermis function as a PPARβ ligand.

## Conclusion

Our results provide new experimental evidence indicating that phytoceramide and yeast-derived sphingoid bases increased transcriptional activities of PPARs. In addition, phytoceramide elevated PPARα activity in hepatic cell lines. These findings suggest that phytoceramide and sphingoid bases derived from fermented foods could be used to regulate the expression of PPAR-targeted genes.

## Abbreviations

cFCS: charcoal-stripped fetal calf serum; CIG: ciglitazone; DHS: dihydrosphingosine; HPLC: high performance liquid chromatography; LBD: ligand binding domain; LD: L-165, 041; M(IP)_2_C: mannosyl-diinositolphosphoryl-ceramide; PHS: phytosphingosine; PPAR: peroxisome proliferator-activated receptor; PPRE: PPAR responsive element; RXR: retinoid × receptor; Sph: sphingosine; UAS: upstream activating sequences; WY: WY-14643.

## Competing interests

The authors declare that they have no competing interests.

## Authors' contributions

IM participated in the design of the study, carried out analysis and interpretation of data and drafted the manuscript. YW helped IM's analysis. SY, TK and KZ carried out lipid extraction. NK and SM participated in the design of the study, contributed to the interpretation of data and revised the manuscript. YM, TS and YI participated in the design of the study and contributed to the interpretation of data. All authors read and approved the final manuscript.
